# Cue-induced Behavioral and Neural Changes among Excessive Internet Gamers and Possible Application of Cue Exposure Therapy to Internet Gaming Disorder

**DOI:** 10.3389/fpsyg.2016.00675

**Published:** 2016-05-09

**Authors:** Yongjun Zhang, Yamikani Ndasauka, Juan Hou, Jiawen Chen, Li zhuang Yang, Ying Wang, Long Han, Junjie Bu, Peng Zhang, Yifeng Zhou, Xiaochu Zhang

**Affiliations:** ^1^Center for Biomedical Engineering, School of Information Science and Technology, University of Science and Technology of ChinaHefei, China; ^2^School of Foreign Languages, Anhui Jianzhu UniversityHefei, China; ^3^School of Humanities and Social Science, University of Science and Technology of ChinaHefei, China; ^4^Department of Philosophy, Anhui UniversityHefei, China; ^5^CAS Key Laboratory of Brain Function and Disease, School of Life Science, University of Science and Technology of ChinaHefei, China; ^6^State Key Laboratory of Brain and Cognitive Science, Institute of Biophysics, Chinese Academy of SciencesBeijing, China; ^7^Center of Medical Physics and Technology, Hefei Institutes of Physical Science, Chinese Academy of SciencesHefei, China

**Keywords:** Internet gaming disorder, excessive Internet gamers, cue-induced changes, substance use disorder, cue exposure therapy, mini-review

## Abstract

Internet gaming disorder (IGD) may lead to many negative consequences in everyday life, yet there is currently no effective treatment for IGD. Cue-reactivity paradigm is commonly used to evaluate craving for substance, food, and gambling; cue exposure therapy (CET) is applied to treating substance use disorders (SUDs) and some other psychological disorders such as pathological gambling (PG). However, no study has explored CET’s application to the treatment of IGD except two articles having implied that cues’ exposure may have therapeutic effect on IGD. This paper reviews studies on cue-induced behavioral and neural changes in excessive Internet gamers, indicating that behavioral and neural mechanisms of IGD mostly overlap with those of SUD. The CET’s effects in the treatment of SUDs and PG are also reviewed. We finally propose an optimized CET paradigm, which future studies should consider and investigate as a probable treatment of IGD.

## Introduction

Internet gaming disorder (IGD), is arguably the most problematic form of Internet use (Petry and O’Brien, 2013) and is different from other forms of problematic Internet use in terms of its prevalence rates, etiologies, characteristics of persons participating in them, and risks for harm (Ko et al., 2007, 2009b; van Rooij et al., 2010). IGD can also lead to comorbid disorders such as major depression, anxiety disorders, attention-deficit hyperactivity disorder, and schizophrenia (Ha et al., 2006). However, there is currently no effective therapy of this disorder.

Recent studies have shown that it is necessary to replace pharmacotherapy with intervention on sensitivity and reactivity to environmental cues in treating substance use disorders (SUDs; Perry et al., 2011). Cue exposure therapy (CET) is such an intervention and is based on Pavlov’s theory of classical conditioning, in which an unconditioned cue (UCS) eliciting an unconditioned response (UCR) is consistently paired with a conditioned cue (CS) until the CS itself can evoke a conditioned response (CR) similar to UCR. The aim of CET is to diminish conditioned relation between an addictive cue (CS) and a physiological response (CR) by systematically pairing them in a treatment setting. Continual combination of a CR with a CS in absence of the actual substance or state it is formerly accompanied with can gradually weaken the CR to the CS, which eventually results in the extinction of the CS–CR association, consequently decreasing reactivity to addictive cues.

Cue exposure therapy is widely used in treating SUDs (Maltby et al., 2002; Hofmann et al., 2006; Costa et al., 2008; Reger and Gahm, 2008; Vögele et al., 2010). However, studies on its use in IGD are minimal. In this paper, we briefly review studies using game-related cues to induce behavioral or neural changes among excessive Internet gamers, and discuss possible adoption of CET in treating IGD.

## Materials and Methods

We searched for literatures on PubMed, Web of Science, and ScienceDirect with no date restrictions. Terms used were “Internet gam^∗^” or “online gam^∗^” or “computer gam^∗^” in combination with “cue^∗^” or “attention^∗^” or “inhibit^∗^” or “neur^∗^”. We finally selected most relevant papers for our review.

### Studies on Cue-induced Behavioral Changes in Excessive Internet Gamers

In substance-related addiction and pathological gambling (PG), two behavioral tendencies associated with addictive disorders are usually studied. The first tendency is attentional bias in which addicted persons unconsciously allocate attention to addiction cues (Field and Cox, 2008), and is often investigated with stroop or dot-probe paradigms (Boyer and Dickerson, 2003; Lusher et al., 2004; Robbins and Ehrman, 2004; Field and Cox, 2008). The second is diminished response inhibition, in which addicted person’s ability to suppress responses to addictive cues is diminished, and is commonly detected using go/no-go task (Goudriaan et al., 2006; Dawkins et al., 2009). Exploring the presence of the two behavioral tendencies in excessive Internet gamers may help us understand behavioral mechanism of IGD and possible therapeutic intervention.

The following three studies focused on both attentional bias and response inhibition toward game-related cues among people with IGD. The first study, by Decker and Gay (2011), used go/no-go task to examine existence of cognitive bias toward gaming-related words in players of World of Warcraft (WoW). The study found that WoW players had higher response disinhibition and cognitive bias toward WoW jargon in all conditions compared with non-players. The second study, by Zhou et al. (2012), used Internet game-shifting task, a variant of go/no-go task, in which game-related pictures and fruit pictures were set as targets and distracters, respectively, or in reverse, and subjects were required to respond to the targets but not respond to the distracters. They found that excessive gamers showed cognitive bias, response inhibition, and shifting deficits toward Internet game-related pictures, and length of addiction (number of years) was positively related to severity of cognitive bias. The third study, by Holst et al. (2012), also showed that higher levels of self-reported problematic gaming were related to more errors on both attentional bias and response inhibition to game cues among male adolescents. These results are consistent with findings of attentional bias reported in clinically recognized SUDs and PG (Boyer and Dickerson, 2003; Robbins and Ehrman, 2004).

Whilst the above three studies investigated both attentional bias and response inhibition, the following three studies focused on one of the two behavioral tendencies. The first study, by Metcalf and Pammer (2011), used a modified Stroop task to investigate the existence of attentional bias for gaming-related words in excessive Massively Multiplayer Online Role-Playing Gamers (MMORPGers). The modified Stroop task comprised of game-related, negative and neutral words presented in different colors and participants were required to indicate the words’ color as quickly as possible. Results showed that excessive MMORPGers had attentional bias toward negative and MMORPG words. The second study, by Liu et al. (2014), examined deficit of response inhibition mechanism in game abusers and explored underlying neurological substrates related to abusers’ implicit cognitive process. Results showed that brain activations of the superior parietal lobe and right dorsolateral prefrontal cortex (DLPFC) were negatively associated with performance of response inhibition among subjects with IGD. Similarly, the third study, conducted by Chen et al. (2014), not only validated the existence of response disinhibition in subjects with IGD, but suggested that dysfunctional activation of the supplement motor area in response disinhibiton is one of the candidate mechanisms in both IGD and SUD.

Not all of the studies exploring cue-induced behavioral changes in subjects with IGD use the terms “attentional bias” or “response inhibition” explicitly. Thalemann et al. (2007) employed the phrase “increased emotional processing” to indicate subjects’ attentional bias and impairment of response inhibition toward addiction cues. They demonstrated that significant between-group differences in cue-induced event-related potentials found at parietal regions pointed to an “increased emotional processing” of these cues in excessive computer game players. The study further concluded that game-related cues had gained an “intrinsic motivational relevance” during learning process for excessive gamers. Yen et al. (2011) used another term “implicit cognition,” a broader category encompassing attentional bias or response disinhibition. It is conceptualized and measured by various cognitive processes in abusers’ performance such as response disinhibition, attentional bias, and memory associations (Stacy and Wiers, 2010; Decker and Gay, 2011). The IGD group responded faster to congruent pairing and had positive motivational implicit response to Internet gaming cues compared to the control group, which highlighted the important role of “implicit cognition” in young adults with IGD.

All the above studies demonstrate that Internet gaming abusers show attentional bias and response disinhibition toward game-related cues or emotional cues, which are considered typical behavioral characteristics of SUD and PG. (Coskunpinar and Cyders, 2013; Hønsi et al., 2013; Smith et al., 2014; Yau and Potenza, 2015).

### Studies on Cue-induced Neural Changes in Excessive Internet Gamers

Neuroimaging studies have found that behavioral and substance addictions share neurocircuitry and that subcortical and frontal cortical areas, especially the ventromedial prefrontal cortex, contribute greatly to neural systems and neurobiological mechanisms of both behavioral and substance addictions (Power et al., 2011; Yau and Potenza, 2015). Existing studies on neurobiological mechanism of SUD and PG have adopted cue-reactivity paradigm, prompting researchers on Internet gaming to employ this paradigm to investigate the neural mechanism of IGD.

In three successive fMRI studies, using different methods and perspectives, Ko et al. (2009a, 2013a,b) concluded that cue-induced neural mechanism of IGD is similar to that of SUD. The first study compared brain activation between Internet game abusers and non-abusers presented with game-related pictures and mosaic pictures. Result showed that right orbitofrontal cortex, right nucleus accumbens, bilateral anterior cingulated and medialfrontal cortex, right DLPFC, and right caudate nucleus, known to relate to craving in SUD, were activated by gaming pictures in gaming abusers. The second study evaluated brain correlates of cue-induced craving to game-related cues in subjects with IGD, subjects in remission from IGD and controls. Results showed that bilateral DLPFC, precuneus, left parahippocampus, posterior cingulate and right anterior cingulate were activated in response to gaming cues in the IGD group and their activation was stronger in the IGD group than in the control group. Furthermore, the IGD group had stronger activation over right DLPFC and left parahippocampus than the remission group. Given that Internet abuse is often comorbid with problematic substance use (Ko et al., 2006, 2008; Yen et al., 2009), Ko et al. (2013b) conducted the third study evaluating brain correlates of cue-induced gaming urge and smoking craving among subjects with both IGD and nicotine dependence. Result showed greater activation in anterior cingulate, parahippocampus, and bilateral parahippocampal gyrus for both smoking craving and gaming urge among the comorbid group than the control group.

Two studies conducted in China further validated the similarity between IGD and SUD in terms of the neural mechanism. Sun et al. (2012) used fMRI to explore craving-related brain regions induced by game associated pictures in Internet game abusers. Results showed that game abusers’ craving was induced by game cue pictures, which caused increased brain activation in some cerebral regions, including bilateral DLPFC, cingulated cortex, right inferior parietal lobule regions, and cerebellum. Increased imaging signal densities were significant and positively correlated with craving scores in the bilateral prefrontal cortex, anterior cingulate cortex and right inferior parietal lobe, all of which are related to cognitive and emotional processing. Given that there is a transition in processing of drug-related cues from the ventral striatum (VS) to the dorsal striatum (DS), Liu et al. (2016) conducted the first fMRI study to explore the function of both VS and DS in response to game-related cues among people with IGD. Results suggested that a transition from VS to DS processing may occur among people with IGD, which is consistent with studies in SUD.

Unlike studies conducted by Ko et al. (2009b, 2013a,b), Sun et al. (2012), and Liu et al. (2016), in which Internet game abusers were placed in one group, Han et al. (2010b, 2011) recruited only healthy subjects in their two cohort studies. The aim of their studies was to investigate cue-induced changes in cortex activity after a certain period of Internet video game play and if such cue presentation could activate similar brain regions observed in people with SUD or PG. The first study suggested that brain activity changes in the frontal-lobe areas after six weeks of extended Internet game play may be similar to those observed in early stages of SUD. In the second study, reported desire in subjects who played more Internet video game (MIGP) positively correlated with brain activation in right medial frontal lobe and right parahippocampal gyrus. The authors concluded that cue-induced activated brain regions in MIGP cohort are similar to those observed in persons with SUD or PG and cues appear to elicit activity in the DLPFC, orbitofrontal cortex, parahippocampal gyrus, and thalamus.

Some studies have suggested that brain activation decreases in response to cues among patients with SUD or PG after antidepressants treatment (Robertson et al., 2007; Chung et al., 2009; Hays et al., 2009). Han et al. (2010a) hence conducted the first bupropion treatment study in patients with IGD to find out if the same effect would be observed in people with IGD. Results showed that, after 6 weeks of bupropion sustained release treatment, both Internet gaming addiction (IGA’s) craving for Internet video game and activity of DLPFC in response to video game cue stimulation decreased, and the former was positively correlated with the latter. The bupropion’s therapeutic effect on IGD in terms of decreasing craving is hence similar to that on SUD or PG.

Based on the above-reviewed studies, activated brain circuit in people with IGD is similar to that in people with SUD. Further, craving for Internet games positively correlats with activated brain regions involved in cognitive and emotional processing. IGD seems to share similar neural mechanism with SUD and PG (Crockford et al., 2005; Goudriaan et al., 2014; Yau and Potenza, 2015).

### Cue Exposure Therapy in the Treatment of SUDs and PG

Cue exposure therapy has been utilized in treating SUDs since 1980s, and appears to be effective in treating SUD although the effect is not significantly superior to other behavioral therapies (Conklin and Tiffany, 2002; Martin et al., 2010; Myers and Carlezon, 2012; Antoine et al., 2014). A meta-analytic review found insufficient evidence to validate CET’s efficacy in treating SUD and the authors highlighted threats to extinction such as renewal, spontaneous recovery, reinstatement, and characteristics of the cues that may weaken the CET effect (Conklin and Tiffany, 2002). An updated review (Martin et al., 2010) found CET as effective in reducing individuals’ craving in some studies. However, the review also obtained little evidence of CET’s superior efficacy over other forms of treatment, although some studies in the review had made innovations including consideration of above-mentioned threats to extinction, attention to individual differences, use of virtual reality (VR) technology, and medication augmentation.

Subsequently, two other reviews focusing on CET combined with medication augmentation (Myers and Carlezon, 2012) and VR technology (Antoine et al., 2014) were conducted. Myers and Carlezon (2012) reviewed studies that examined the effect of D-cycloserine (DCS) on extinction of cue-induced conditioned responses in SUD. They concluded that existing data indicated less robust effects of DCS-coupled CET in SUD. Since medication augmentation measures bear some limitations, some neuromodulation techniques such as repetitive transcranial magnetic stimulation (rTMS) and transcranial direct current stimulation (tDCS) are more favorable to augment CET effect. Similarly, studies combining CET with VR have also reported mitigated efficacy in treating SUD (Antoine et al., 2014). However, this review indicated that VR can improve actual assessment of craving in SUDs and can significantly help increase ecological cue validity so as to facilitate VR–CET efficacy at individual level. VR technology may have advantages if used in treating IGD because of its simulation of either the scenario of Internet game or the environments in which abusers play games, which can bring about subjects’ visual stimulation as experienced in their gaming condition. Meanwhile, VR–CET can increase game-related cue’s ecological validity and fulfill individualized treatment, which cannot be realized by standard cue-induced paradigms using common words, pictures, or videos.

A recent study explicitly confirmed CET’s efficacy in treating SUD (Xue et al., 2012). This study reported a memory retrieval-extinction procedure that successfully decreased cue-induced craving in abstinent heroin abusers 1, 30, and 180 days later, and concluded that in extinction sessions, cue exposure can only work within a specific time interval termed “reconsolidation window” (Tronson and Taylor, 2007; Nader and Hardt, 2009) which is up to 2 h after memory retrieval.

Besides, results from studies adopting CET in treating PG also showed CET has its potential in the reduction or extinction of gambling urges. Two case studies adopting CET successfully reduced subjects’ gambling behavior and urges (Symes and Nicki, 1997). Kushner et al. (2007) suggested that problem gamblers’ intensity of gambling urges decreases with time when exposed to gambling cues, especially in the presence of gambling-relevant negative mood induction manipulation. Another study validated the use of VR in cue exposure, and showed that a number of exposure sessions are essential to trigger the extinction process (Giroux et al., 2013).

### Cue’s Possible Therapeutic Effect on IGD

To our knowledge, few studies have used standard CET paradigm to treat IGD and only two studies reviewed below indicate cue’s possible therapeutic effect on IGD.

Lorenz et al. (2013) conducted an fMRI study using a dot probe paradigm with short-presentation (SP) trial used for investigating subjects’ attentional bias and long-presentation (LP) trial used for investigating subjects’ cue reactivity in eight male pathological computer game players (PCGPs) and nine healthy controls. The study found that PCGPs showed an attentional bias toward both game-related and affective stimuli with positive valence. Furthermore, activation brain areas in PCGPs were different between SP trial and LP trial, in which cue-induced brain responses can be inhibited by top–down inhibitory processes associating with the right inferior frontal gyrus. This implies that such cognitive inhibition processes in cue reactivity might be essential in therapy for the treatment of IGD. Kim et al. (2013) investigated the influence of a course with narrative characteristics and content borrowed from a MMORPG on game abusers’ language expression and the course’s therapeutic effect on gaming disorder. Analysis of P300 amplitude and sLORETA images before and after the course showed that game abusers had a processing bias toward game-related cues before the course and such bias was weakened after the course. Thus, game abusers’ mental processing used for the course was changed and their craving decreased.

The above-mentioned two studies already take on some features of CET and the findings bear some implications for future CET’s application in treating IGD. Lorenz et al. (2013) highlighted the possibility of inhibiting cue-induced brain responses and the importance of cognitive inhibition processes arising in cue’s LP trial lasting for 2000 ms. This implies that there is a possible duration threshold for presenting cues in CET so that cognitive inhibition processes can take place. In the two-month study, Kim et al. (2013) used different forms of cues such as video game clips, images of game scenes, and written form of game story. Therefore, longer duration of study and more types of cues are worth consideration in future studies employing CET as treatment of IGD.

## Conclusion and Future Study

In view of CET’s efficacy in decreasing craving for substance-related cues, its extinction of cue-response association (Antoine et al., 2014) and the overlaps between IGD and SUD with regard to their neural and behavioral mechanisms, we suggest taking more studies on the effect of applying CET paradigm to the treatment of IGD. Given that CET’s efficacy in treating SUD is overall mitigated, optimizing the traditional CET paradigm to ensure a better therapeutic effect is essential for future studies. Combining VR–CET with tDCS or conducting tDCS after VR–CET sessions may help ensure a long term effect in treating IGD. Further, when adopting CET paradigm in treating IGD, future studies should validate whether conducting the extinction process within the “reconsolidation window” can also help to weaken or even erase the original game-cue memory. Besides, some methodological problems such as threats to extinction, permission or forbiddance of online gaming between CET sessions, participants’ individual differences, small sample size and high dropout rates should be considered so as to strengthen CET efficacy. If the proposed optimized CET paradigm, namely VR–CET coupled with tDCS, can work and bear long-term therapeutic effect, then it would be undoubtedly a more favorable treatment of IGD.

## Author Contributions

XZ supervised this study and revised each draft. YZ wrote this paper. YN proofread each draft. JH and LY provided suggestions on the structure of this paper. JC, YW, LH, JB, PZ, and YZ helped to collect the materials and resources needed for this study.

## Conflict of Interest Statement

The authors declare that the research was conducted in the absence of any commercial or financial relationships that could be construed as a potential conflict of interest.
